# The phytochemical arbutin exerts potent anti-*Toxoplasma* effects through activation of cell-autonomous defense mechanisms while attenuating inflammation

**DOI:** 10.1371/journal.pntd.0013815

**Published:** 2025-12-11

**Authors:** Zhuanzhuan Liu, Yulu Ma, Yaoyao Xiang, Yusi Shen, Longxiang Liao, Xiuwen Zhu, Zhen Shi, Yanxia Wei, Yanbo Kou, Yugang Wang

**Affiliations:** 1 Jiangsu Key Laboratory of Immunity and Metabolism, Xuzhou Medical University, Xuzhou, China; 2 Laboratory of Infection and Immunity, Department of Pathogenic Biology and Immunology, School of Basic Medical Sciences, Xuzhou Medical University, Xuzhou, China; Universidade Federal de Minas Gerais, BRAZIL

## Abstract

**Background:**

Plant-derived natural products have emerged as promising candidates for developing novel anti-toxoplasmosis drugs. This study aimed to elucidate the role and mechanism of the phytochemical arbutin in the control of *T. gondii* infection.

**Methodology/Principal findings:**

The effect of arbutin on *T. gondii* infection and host inflammatory response was evaluated both in vitro and in vivo. RNA-seq was performed on mouse bone marrow-derived macrophage samples to identify potential arbutin-related biological processes and molecular targets that control *T. gondii* infection. These targets were further confirmed using target-specific activators or inhibitors. Our data indicated that arbutin has dual therapeutic effects against *T. gondii* infection through concurrently controlling parasite growth and mitigating infection-induced inflammation. Mechanistically, arbutin mediates restriction of intracellular labile iron pool in both immune and non-immune cells, thereby depriving the parasite of essential metal nutrients. In addition, in macrophages, arbutin not only inhibits infection-induced inflammatory response but also upregulates the expression of heme degrading enzyme heme oxygenase-1, which facilitates biliverdin production. Our data further demonstrated that biliverdin exhibits anti-*T. gondii* effector function. Furthermore, arbutin is also effective in reducing infection-related mortality in immunocompromised mice.

**Conclusions/Significance:**

Our data highlight arbutin’s potential therapeutic value in fighting against acute hyperinflammatory phase of Toxoplasmosis even in immunocompromised host but also its limitation in establishing long-term immunity. Our study further suggests a potential direction for further development of effective drugs to prevent and treat toxoplasmosis by pharmacologically enhancing cell-autonomous defense mechanisms while suppressing inflammatory response.

## Author summary

Current *Toxoplasma gondii* therapies poorly balance parasite clearance and damaging inflammation. We show the phytochemical arbutin offers a dual-action solution. It restricts parasite growth in diverse cells by depleting labile iron pool essential for *T. gondii*. Simultaneously, arbutin attenuates infection-induced inflammation. In macrophages, it uniquely upregulates heme oxygenase-1 (HMOX1), producing biliverdin which directly kills parasites. Arbutin significantly improves survival in mouse models of infection, including immunocompromised hosts, while reducing inflammation. This demonstrates how enhancing cell-autonomous defense via metabolic intervention can effectively control toxoplasmosis and immunopathology.

## Introduction

*Toxoplasma gondii* (*T. gondii*, Tg) is a protozoan parasite belonging to the phylum Apicomplexa and can invade almost all warm-blooded vertebrates, including humans, causing zoonotic disease toxoplasmosis. Serological evidence indicates approximately one-third of the global human population is infected with Tg [1]. Host immunity is essential for the control of toxoplasmosis progression. However, immune response to Tg infection is a double-edged sword: while essential for parasite control, excessive inflammation can drive immunopathology including intestinal ileitis and encephalitis [2,3]. This creates a therapeutic dilemma: eliminating the parasite while preventing immune-mediated tissue damage. How to resolve this paradox remains to be a significant clinical challenge.

Current therapeutic strategies for toxoplasmosis remain limited to a small repertoire of drugs, most of which have severe side effects, and seeking ideal therapeutic agents remains to be a long-term and challenging mission [4]. Plant-derived natural products have emerged as promising candidates for developing novel anti-toxoplasmosis drugs. Arbutin (4-hydroxyphenyl-β-D-glucopyranoside, β-arbutin), a naturally occurring β-D-glucopyranoside derivative of hydroquinone primarily extracted from the leaves of bearberry plant *Arctostaphylos uva-ursi*, exhibits multifaceted bioactivities including antioxidant, anti-inflammatory, antimicrobial, antihyperglycemic, and antitumor properties [5]. Arbutin has a spatial isomer, α-arbutin (4-hydroxyphenyl-α-D-glucopyranoside), which is a synthetic compound. Both isomers act as competitive inhibitors of tyrosinase, the key enzyme involved in melanin biosynthesis. Although both compounds exhibit skin-lightening properties, α-arbutin demonstrates superior efficacy compared to its β-form counterpart. Clinically, arbutin is used in drugs for treatment of lower urinary tract infections and as a food supplement, whereas α-arbutin is primarily used as a dermatological depigmenting agent [6]. Preliminary observational studies suggest anti-*Toxoplasma* activity of arbutin isolated from *Orostachys malacophylla* (Green Duncecap) or *Pyrus boissieriana* (Boissier Pear) [7,8], but its precise mechanism of action remains enigmatic. In this study, we aimed to explore the potential mechanisms of arbutin-mediated anti-*T. gondii* activity both in vitro and in vivo.

## Methods

### Ethics statement

All animal experiments were approved by the Institutional Animal Care and Use Committee (IACUC) of Xuzhou Medical University (Xuzhou, China; SCXK (Su) 2020–0048).

### Animals

Six-week-old male C57BL/6 wild-type (WT) mice were obtained from GemPharmatech Co., Ltd. (Nanjing, Jiangsu, China), Kunming WT mice were from Xuzhou Medical University experimental animal center, and nude mice from Cavens (Changzhou, Jiangsu, China). All animals were maintained under specific pathogen-free (SPF) conditions in a temperature-controlled environment (25 ± 2°C) with a 12-hour light/dark cycle. The mice had free access to food and water.

For *T. gondii* (Tg) infection, each mouse was intraperitoneally injected with 2 × 10³ tachyzoites of the Tg RH strain or orally gavaged with 50 cysts of the *T. gondii* Chinese 1 genotype Wh6 (TgCtwh6) strain (both are kindly provided by Professor Jilong Shen from Anhui Medical University, Hefei, China) [9]. Three days prior to infection, the drinking water was supplemented with either 0 mg/mL, 5 mg/mL, or 50 mg/mL arbutin, and this treatment continued throughout the experiment. Giemsa staining for tachyzoites in the peritoneal fluid was performed using a solution from Solarbio (Beijing, China). For serum cytokine measurement, we used a Cytometric Bead Array (CBA) mouse inflammation kit from BD Biosciences (Franklin Lakes, NJ, US).

A normal control diet (40 ppm Fe) or a high-iron diet (1000 ppm Fe) is based on the AIN-93G formulation (Research Diets, Beijing, China). Biliverdin-supplemented custom diet (20 mg/kg) and its control diet were made by Beijing Keao Xieli Feed Co., Ltd. (Beijing, China), and the diet was stored at 4°C and kept away from light.

To establish a model of pharmacologically induced immunosuppression that mimics clinical conditions such as chemotherapy, mice were treated with 2 mg/mouse cyclophosphamide (CTX, purchased from MCE, NJ, USA) intraperitoneally once every week, starting from day -7 prior to infection.

### Cell lines and *T. gondii* strains

Human foreskin fibroblast cells (HFF, ATCC-SCRC-1041) were cultured in Dulbecco’s Modified Eagle Medium (DMEM) supplemented with 10% fetal bovine serum (FBS), 1% penicillin-streptomycin, and 1% 1 M 4-(2-Hydroxyethyl)-1-piperazineethanesulfonic acid (HEPES) (Solarbio, Beijing, China). Human leukemia monocytic cell line THP-1 cells (ATCC TIB-202) were maintained in Roswell Park Memorial Institute (RPMI)-1640 medium supplemented with 10% FBS and 1% penicillin-streptomycin. THP-1 cells were differentiated into adherent macrophages by treatment with 100 ng/mL phorbol 12-myristate 13-acetate (PMA) (MCE, NJ, USA) for 24 h and subsequently used for further experiments. β-Arbutin (CAS#: 497-76-7) was purchased from Aladdin Scientific (Shanghai, China). For iron supplementation, THP-1 and HFF cells were pretreated with 10 mM ferric ammonium citrate (FAC) from MCE (NJ, USA) or 50 µM FeSO4 (Macklin, Shanghai, China) for 12 h, respectively, before infection with Tg RH tachyzoites. For ferroportin (FPN) inhibition, THP-1 and HFF cells were pretreated with 20 μM VIT-2763 (MCE, NJ, USA) for 2 h before infection with Tg RH tachyzoites. For heme oxygenase-1 (HMOX1) induction and activation, bone marrow derived macrophages (BMDMs) were pretreated with hemin (10 μM or 40 μM, MCE, NJ, USA) 2 h before infection. Biliverdin were purchased from Sigma (MO, USA). CORM-3 and Bilirubin were from MCE (NJ, USA).

Tachyzoites of the *T. gondii* RH strain was propagated as previously described [9]. Cysts of the Wh6 strain were isolated from the brain homogenates of *T. gondii* Wh6-infected Kunming mice, as described previously [10]. For in vitro infection, the multiplicity of infection (MOI) for RH tachyzoites was 1:1 for BMDMs and THP-1 cells, and 1:5 for HFF cells. The parasites were incubated with the cells for 30 min, after which the uninvaded tachyzoites were washed away.

### Isolation of bone marrow derived macrophages (BMDMs)

The BMDMs were isolated from the femurs and tibias of wild-type C57BL/6 mice, following a previously described protocol [9].

### Cell viability (CCK-8) assay

The cell viability was determined using a CCK-8 assay kit (Meilunbio, Dalian, Liaoning, China). THP-1 cells were seeded in a 96-well plate at a density of 1 × 10^5^ cells per well and cultured for 24 h. Arbutin was added 12 h prior to infection with RH tachyzoites at MOI of 1:1. After 24 h post-infection, the culture medium was replaced with a CCK-8 solution, and the plate was incubated at 37°C for 1 h. Absorbance was measured at 450 nm using a microplate reader. The cell survival rate was calculated according to the following formula:

Relative Cell Viability (%) = (OD_Experimental Group_ - OD_Blank Group_)/ (OD_Control Group_ - OD_Blank Group_) × 100%.

### The relative parasite burden determination

The genomic DNA from the tissue or cell samples was extracted using a DNA extraction kit provided by Tiangen Biotech Co., Ltd. (Beijing, China). All DNA samples were quantified and assessed for purity using NanoDrop2000 spectrophotometer. The relative parasite burden was subsequently quantified via quantitative PCR (qPCR) by measuring the relative DNA copy number of the *T. gondii* surface antigen 1 (Tg*Sag1*) gene as a proxy. The following primer sequences were utilized: for Tg*Sag1*, 5’-GTCGTTCTTGCGATGTGG-3’ (forward) and 5’-TTTGCCTGTTGGGTGAGTA-3’ (reverse); for mouse *β-actin* (m*β-actin*), 5’-TGAGAGGGAAATCGTGCGTGAC-3’ (forward) and 5’-GCTCGTTGCCAATAGTGATGACC-3’ (reverse); and for human glyceraldehyde-3-phosphate dehydrogenase (h*Gapdh*), 5’-GAAGGTGAAGGTCGGAGTC-3’ (forward) and 5’-GAAGATGGTGATGGGATTTC-3’ (reverse).

### Immunofluorescence

BMDMs or THP-1 cells were fixed with 4% paraformaldehyde (Servicebio, Wuhan, Hubei, China) for 20 min at room temperature, and permeabilized using 0.1% Triton X-100 (Vicmed, Xuzhou, Jiangsu, China) diluted in PBS for 20 min on ice. After blocking with 3% bovine serum albumin (BSA) (Wigedbio, Xuzhou, Jiangsu, China), the cells were stained with an anti-TgSAG1 monoclonal antibody (Invitrogen, Carlsbad, CA, USA) diluted 1:100 in 0.1% Triton X-100 at 4°C overnight. Secondary antibody staining was performed at room temperature for 1 hour using a CoraLite 594-conjugated goat anti-mouse IgG (H + L) antibody (Proteintech, Wuhan, Hubei, China) diluted 1:1000 in 0.1% Triton X-100. The cells were then stained with FITC-phalloidin (Abclonal, Wuhan, Hubei, China) diluted 1:100 in 0.1% Triton X-100 at room temperature for 1 h, followed by DAPI (Servicebio, Wuhan, Hubei, China) nuclear staining for 5 min. Finally, after adding antifade mounting medium (MCE, NJ, USA), images were captured using an Olympus IX73 inverted microscope (Tokyo, Japan).

### RNA sequencing

WT BMDMs were pretreated with vehicle or 20 mM arbutin for 12 hours. Cells were then exposed to Tg RH strain tachyzoites (MOI = 1) or left uninfected. At 4 hours post-infection, cells were harvested for total RNA extraction using TRIzol Reagent according the manufacturer’s instructions. The RNA quality was determined by 5300 Bioanalyser (Agilent, Santa Clara, CA, USA) and quantified using the ND-2000 (NanoDrop Technologies, Wilmington, DE, USA). The RNA-seq transcriptome library was prepared following Illumina (San Diego, CA) Stranded mRNA Prep, Ligation protocol using 1μg of total RNA, which utilizes poly(A) selection to enrich polyadenylated mRNA from total RNA. This approach efficiently depletes rRNA, thereby maximizing mRNA sequencing depth and ensuring high-quality transcriptome data. The RNA-seq data are deposited in the NCBI Sequence Read Archive (SRA) under the accession number PRJNA1108606.

### Real-time quantitative reverse transcription PCR (qRT-PCR) analysis

Total RNA was isolated using TRIzol reagent (Vicmed, Jiangsu, China). All samples were quantified and assessed for purity using a spectrophotometer (NanoDrop2000). The A260/A280 ratios for all samples were between 1.8 and 2.0. The residual genomic DNA was removed using gDNA digester Mixture. cDNA was prepared by reverse transcription using the cDNA synthesis kit (Yasen, Shanghai, China). mRNA transcription level was quantified using Hieff qPCR SYBR Green Master Mix No Rox (Yeasen, Shanghai, China) on a Roche LightCycler 480. The following primers were used: **m*Il-1β***, 5’-TGGCAACTGTTCCTG-3’ (forward) and 5’-GGAAGCAGCCCTTCATCTTT-3’ (reverse); **m*Il-6***, 5’-TAGTCCTTCCTACCCCAATTTCC-3’(forward) and 5’-TTGGTCCTTAGCCACTCCTTC-3’ (reverse); **m*Nos2*,** 5’-CCAAGCCCTCACCTACTTCC-3’ (forward) and 5’-CTCTGAGGGCTGACACAAGG-3’ (reverse); **m*β-actin*,** 5’-TGAGAGGGAAATCGTGCGTGAC’(forward) and 5’-GCTCGTTGCCAATAGTGATGACC-3’ (reverse). The relative transcription levels of the target genes were calculated using the 2^–ΔΔCt^ method, with normalization to the internal reference genes (m*β-actin*).

### Quenchable iron pool (QIP) assay

Cells that were treated under different conditions were harvested and stained with 1 μM Calcein-AM (BioLegend, San Diego, CA, USA) diluted in PBS for 15 mins at 37 °C, then treated with PBS or FeHQ solution (5 μM FeCl_2_ and 10 μM 8-hydroxyquinoline diluted in PBS) for 30 mins at 37 °C. Cells were then resuspended in FACS buffer (0.1% BSA diluted in PBS). The mean fluorescence intensity (MFI) of Calcein staining was detected by flow cytometry. The QIP value was calculated as the difference in MFI (Calcein) between PBS and FeHQ-treated cells [9].

### Western blot analysis

The following primary antibodies were used for immunoblot: anti-HMOX1 (cat no.,10701–1-AP), anti-FPN1 (cat no.,26601–1-AP), and anti-β-actin (cat no.,66009–1-Ig) were purchased from Proteintech (Wuhan, China); anti-α-tubulin (cat no., YM3035) was purchased from ImmunoWay Biotechnology (Newark, NJ, USA); anti-TFR1 (cat no.,13113T) was purchased from Cell Signaling Technology (Danvers, MA, USA).

### RNA interference

The specific small interfering RNA (siRNA) targeting human *HMOX1* and scramble siRNA negative control were purchased from GenePharma (Shanghai, China). Targeted sequences for *HMOX1* were as follows: 5’-CCAGCAACAAAGUGCAA GATT-3’ (forward);5’-UCUUGCACUUUGUUGCUGGTT-3’ (reverse). THP-1 cells were transfected with siRNA using Lipofectamine 3000 (ThermoFisher, Waltham, MA, USA) according to the manufacturer′s protocol. After 6 h transfection, basal medium was replaced with complete medium and incubated for another 48 h, followed by Tg RH infection (MOI = 0.25). Cells were harvested 24 hpi to determine the intracellular parasite load.

### Evaluation of immunosuppressive effect of cyclophosphamide (CTX) by flow cytometry

Kunming WT mice received two intraperitoneal injections of CTX doses (100 mg/kg) at 7-day intervals. Peripheral whole blood collected at different time-points before and after CTX injection was used to detect lymphocytes and their subsets by flow cytometry. The following antibodies were used: FITC anti-mouse CD45.2 (BioLegend, San Diego, CA, USA) (at a dilution ratio of 1:400), PerCP-Cyanine5.5 anti-mouse CD4 (Tonbo Biosciences, San Diego, CA, USA) (at a dilution ratio of 1:400), PE anti-mouse CD8 (BioLegend, San Diego, CA, USA) (at a dilution ratio of 1:400), and APC-Cy7 anti-mouse CD19 (115530, BioLegend, San Diego, CA, USA) (at a dilution ratio of 1:400).

### Determination of iron ion chelating capacity

A spectrophotometric method was employed to determine the iron ion chelating capacity. Standard solutions of EDTA (Vicmed, Xuzhou, Jiangsu, China) were prepared in ddH_2_O at final concentrations of 100, 200, 400, 800, and 1000 μg/mL. Arbutin samples were prepared in ddH_2_O to achieve concentration gradients of 5, 10, 20, and 50 mM. For the assay, 800 μL of each standard, sample, or control (ddH_2_O) was mixed with 400 μL of the FeCl_2_ solution (600 μg/mL). The mixture was incubated at 25°C for 10 min. Then, 1.5 mL of the sodium acetate buffer (0.2 M, pH 5.6), containing 1% gallic acid (Aladdin Scientific, Shanghai, China), was added, followed by incubation for 10 min at 25°C. The absorbance of the solution was measured at 570 nm using a microplate reader. The percentage of iron ions chelating (PIC%) was calculated according to the following formula: PIC% = [(AC_570_ − AS_570_/ AC_570_] × 100.

AC_570_ represents the absorbance of the control at 570 nm, and AS_570_ represents the absorbance of the standard or sample. A standard curve was plotted using the PIC% of the EDTA standards, which exhibited a linear regression with R^2^ > 0.99.

### Statistical analysis

The statistical analysis of the data was conducted using GraphPad Prism Version 8.0.1 software. The normality of the data was visually examined via the QQ plot. Statistical significance was determined using the two-sided unpaired t-test (for normally distributed data) or Mann-Whitney U test analysis for single variables and one-way or two-way ANOVA analysis for multiple variables. Log-rank (Mantel-Cox) test was used for survival analysis. *P* < 0.05 was considered statistically significant.

## Results

### Arbutin has potent anti-*T. gondii* activities both in vitro and in vivo

**Fig 1:**
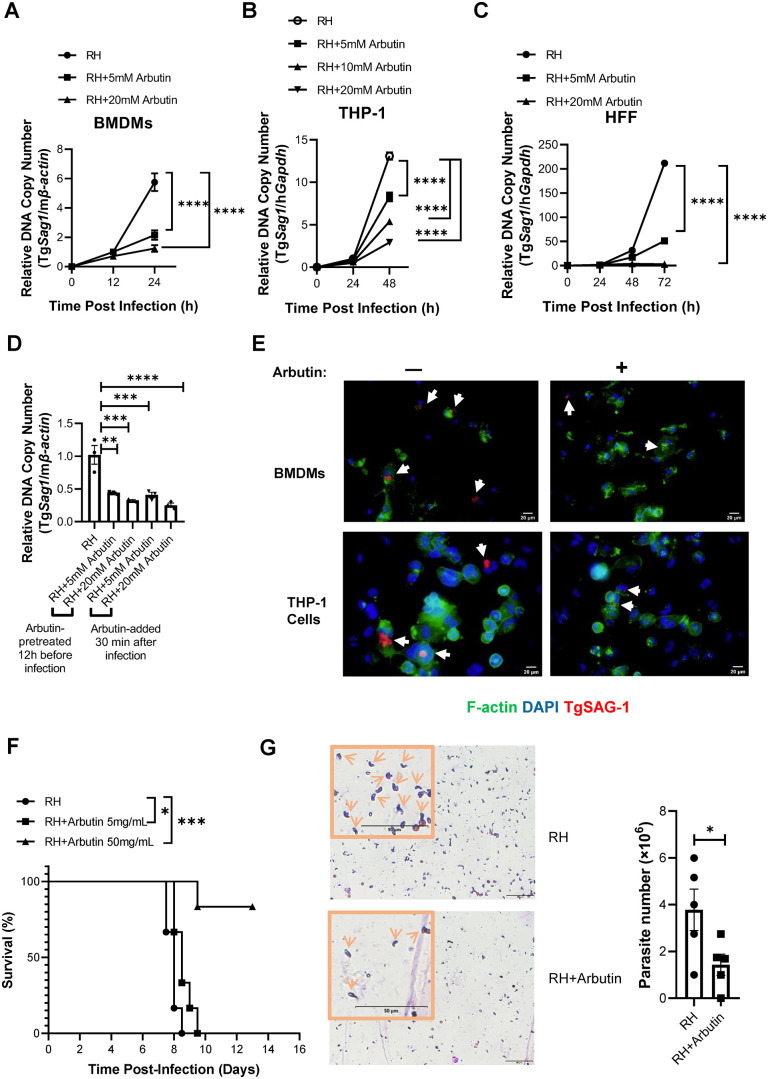
Arbutin has potent anti-*T. gondii* activities both in vitro and in vivo. **(A–C)** Relative intracellular parasite numbers in mouse WT BMDMs (A), human THP-1-derived macrophages (B), or human HFF cells (C) that pretreated with vehicle control or arbutin at the indicated concentrations were quantified by qPCR at the indicated time points post-infection. n = 3 samples per time point. **(D)** The relative intracellular parasite numbers in WT BMDMs that were either pre-treated with arbutin for 12 h before Tg infection, or post-treated with arbutin 30 min after infection, were monitored by qPCR 24 h post-infection. n = 3 samples/group. **(E)** Representative immunofluorescence images of RH tachyzoites in the presence (+) or absence (-) of arbutin pretreatment in the indicated cell types. White arrows indicate the intracellular parasitophorous vacuoles of *Toxoplasma*. Red: TgSAG1; Green: F-actin; Blue: nuclei. Scale bar = 20 μm. n = 3 samples/group. **(F and G)** C57BL/6 WT mice were provided with either vehicle or arbutin-supplemented drinking water three days prior to intraperitoneal infection with Tg RH strain tachyzoites, and they continued to receive the same type of water thereafter. **(F)** The survival rate post infection over time. n = 6 mice/group. **(G)** The number of RH tachyzoites in the peritoneal fluid was quantified by microscopic examination using Giemsa staining eight days post-infection. The inset provides an enlarged view of a portion of the graph, highlighting typical tachyzoites (indicated by arrows). Scale bar = 50 μm. n = 5 mice/group. All of the experiments were repeated at least 2 times. Data are presented as the mean ± SEM. Statistical analysis was performed using one-way ANOVA (D), two-way ANOVA (A–C), Log-rank test for (F), and two-sided Student’s t-test for (G). *P < 0.05; **P < 0.01; ***P < 0.001; ****P < 0.0001.

*T. gondii* (Tg) is an obligate intracellular parasitic protozoan capable of infecting most warm-blooded vertebrates, including humans, and can invade almost any nucleated cells within the host. To investigate the effect of arbutin on Tg infection, we pretreated innate immune cells—specifically, mouse BMDMs or human-derived macrophages generated by treating monocytic THP-1 cells with PMA—and non-immune cells, such as human foreskin fibroblast (HFF) cells, with varying concentrations of arbutin for 12 h. Subsequently, these cells were infected with the Tg RH strain tachyzoites for 30 min. After washing away the uninvaded tachyzoites, arbutin was reintroduced to the cells. We then monitored the parasite load over time. Arbutin significantly inhibited intracellular parasite growth in a concentration-dependent manner across all tested cell types (Fig 1A–1C).

To evaluate whether arbutin also exerts a therapeutic effect after infection is established, we added it to cells post-infection. Arbutin still effectively inhibited the intracellular growth of Tg RH (Fig 1D). The result demonstrates that arbutin possesses both preventative and therapeutic activity against *T. gondii* infection.

Immunofluorescence staining with Tg surface antigen 1 (TgSAG1) demonstrated that arbutin pretreatment resulted in a lower number of infected cells. In the cells that were infected, the TgSAG1-positive puncta were consistently smaller compared to those in the control cells (Fig 1E). Therefore, our data support the conclusion that arbutin is an effective anti-*T. gondii* agent for both immune and non-immune cells, at least in vitro.

To investigate whether arbutin can prevent Tg infection in vivo, C57BL/6 WT mice were provided with either vehicle or arbutin-supplemented drinking water for three days prior to intraperitoneal infection with Tg RH strain tachyzoites, and they continued to receive the same type of water thereafter. Arbutin treatment improved the survival of the mice post-infection in a dose-dependent manner (Fig 1F) and reduced the peritoneal parasite load (Fig 1G). Therefore, arbutin is also effective in preventing Tg infection in mice.

### Arbutin attenuates *T. gondii* infection-induced inflammatory responses

**Fig 2:**
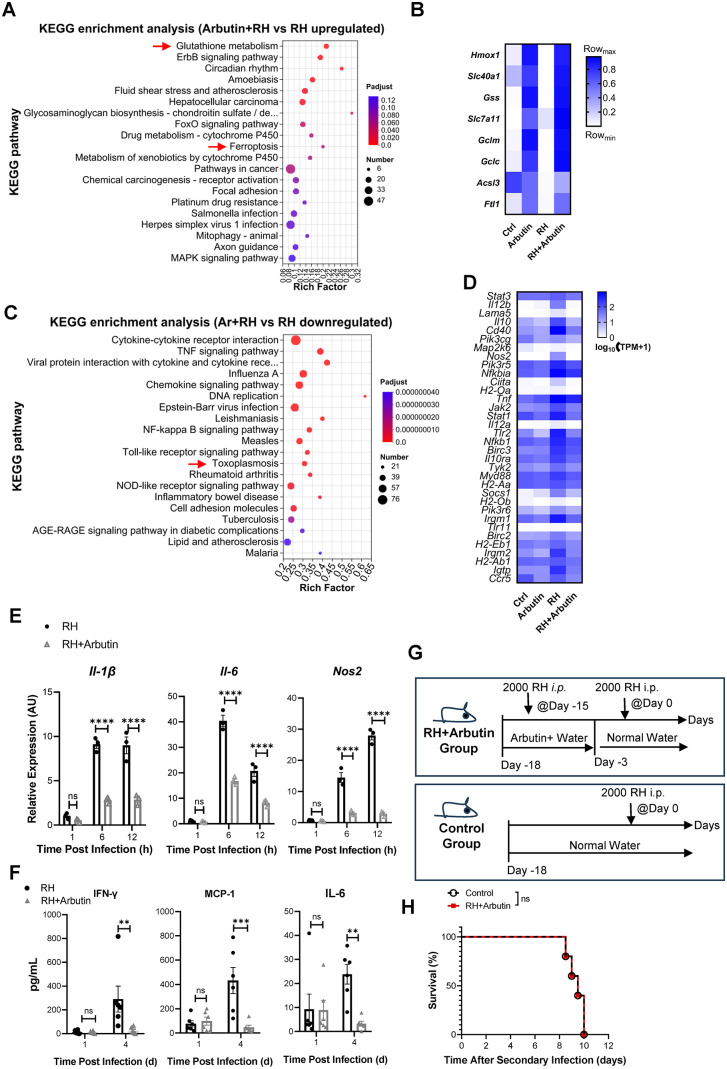
Arbutin attenuates *T. gondii* infection-induced inflammatory responses. **(A–D)** WT BMDMs were pretreated with vehicle or 20 mM arbutin for 12 hours, followed by Tg RH infection or left uninfected. At 4 hours post-infection, cells were harvested for RNA sequencing. “Ctrl”: vehicle, no infection; “Arbutin”: arbutin, no infection; “RH”: vehicle + infection; “RH+Arbutin”: arbutin + infection. n = 3 biological replicates. **(A and C)** KEGG pathway enrichment analysis (adjusted *p* < 0.05) of **(A)** upregulated and **(C)** downregulated pathways in RH + Arbutin vs. RH groups. Top 20 KEGG pathways ranked by enrichment score are displayed. Red arrows highlight the pathways that are related to glutathione metabolism, iron homeostasis, or immune pathways against toxoplasmosis. **(B)** Heatmap visualization of arbutin-induced upregulated genes associated with iron homeostasis and glutathione metabolism. **(D)** Heatmap depicting arbutin-mediated downregulated genes involved in host immune responses against toxoplasmosis. **(E)** Relative mRNA transcription levels of *Il-1β*, *Il-6*, and *Nos2* in WT BMDMs that were pretreated with vehicle control or 5 mM arbutin for 12 h before infection with Tg RH. n = 3 biological replicates per group. **(F)** C57BL/6 WT mice were administered with either vehicle control or arbutin-supplemented drinking water for consecutive days prior to intraperitoneal challenge with Tg RH strain tachyzoites. Serum levels of IFN-γ, CCL2/MCP-1, and IL-6 were measured at 1- and 4-day post-infection. n = 6 mice/group. **(G and H)** Secondary challenge experiment: (G) Schematic diagram of the experimental timeline, including arbutin treatment, primary infection, and secondary challenge phases; (H) Survival rates were monitored daily for 10 days post-secondary infection. n = 5 mice/group. Data were shown as the mean ± SEM. Statistical analysis with two-way ANOVA analysis (E, F), and Log-rank test for (H). **P < 0.01; ***P < 0.001; ****P < 0.0001; ns, no statistical significance.

Macrophages play an important role in the early immune response against *Toxoplasma* and are one of the cell types preferentially infected by the parasite in vivo. To gain a more comprehensive understanding of the effects of arbutin treatment on macrophage function, wild-type BMDMs were pre-treated with either a vehicle control or 20 mM arbutin for 12 h. Subsequently, these cells were exposed to tachyzoites of the Tg RH strain (MOI = 1) or left uninfected. Four hours post-infection, the cells were harvested for RNA sequencing. KEGG (Kyoto Encyclopedia of Genes and Genomes) pathway analysis, based on all differentially expressed genes, revealed that genes involved in regulating cellular iron homeostasis (e.g., *Hmox1*, *Slc40a1*) and glutathione metabolism (e.g., *Gclc*, *Gss*) were upregulated in the RH + arbutin group compared to the RH group (Fig 2A and 2B). Many immunity-related genes associated with host fighting against toxoplasmosis were downregulated (Fig 2C and 2D). Additionally, key genes implicated in the control of Tg infection, such as *Il-1β* (encoding interleukin-1β), *Il-6* (encoding interleukin-6), and *Nos2* (encoding inducible nitric oxide synthase), were also found to be reduced in the arbutin-treated group by qRT-PCR (Fig 2E).

In vivo, arbutin inhibited the systemic inflammatory response induced by Tg infection, as evidenced by reduced serum levels of IFN-γ, CCL2/MCP-1, and IL-6 four days post-infection (Fig 2F). Furthermore, although arbutin protected the host from the lethality associated with primary Tg infection, the withdrawal of arbutin left the surviving animals vulnerable to secondary infection (Fig 2G and 2H), suggesting that arbutin treatment does not induce an effective adaptive immune memory.

### The anti-*T. gondii* effect of arbutin is associated with its capacity to restrict labile iron pool

**Fig 3:**
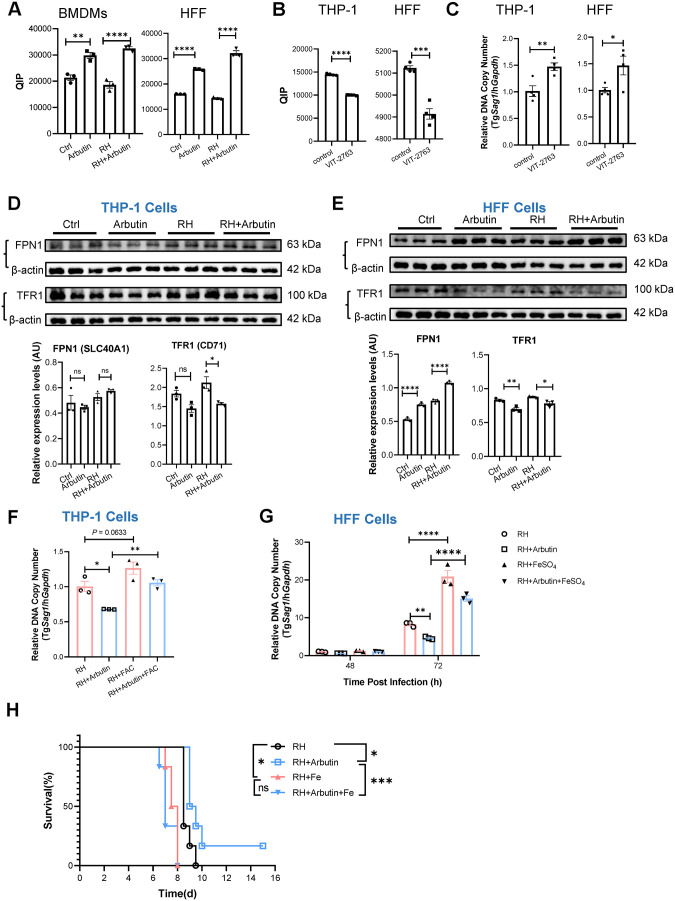
The anti-*T. gondii* effect of arbutin is associated with its capacity to restrict LIP. **(A)** The levels of LIP in BMDMs at 24 hours post-infection (hpi) and in HFF cells at 72 hpi were determined using QIP analysis. “Ctrl” (vehicle, no infection), “Arbutin” (arbutin, no infection), “RH” (vehicle + infection), and “RH+Arbutin” (arbutin + infection). n = 3 samples/group. The experiments were repeated at least 2 times. **(B and C)** The levels of LIP (B) and the relative intracellular parasite numbers (C) were determined in THP-1 cells at 24 hpi and in HFF cells at 72 hpi. Both cell types were pretreated with vehicle control or the FPN inhibitor VIT-2763 for 2 h in the presence of arbutin before infection. n = 4 samples/group. The experiments were repeated at least 2 times. **(D and E)** Western blot analysis and densitometry quantification were performed to assess the protein expression levels of FPN1 and TFR1 in THP-1 cells (D) at 24 hpi and in HFF cells (E) at 72 hpi. n = 3 samples/group. **(F and G)** The parasite loads were determined in THP-1 cells at 24 hpi (F) and in HFF cells at 48 and 72 hpi (G). The cells were pre-treated with either culture medium or arbutin in the presence of FAC (10 mM, for THP-1 cells) or FeSO_4_ (50 μM, for HFF cells), respectively, for 12 hours, followed by exposure to Tg RH tachyzoites. n = 3 samples/group. The experiments were repeated at least 2 times. **(H)** The survival rate post infection was monitored daily in C57BL/6 WT mice that were fed a high-iron (1000 ppm) or a normal iron control diet (40 ppm) starting on day -7 before infection. n = 6 mice/group. Data were shown as the mean ± SEM. Statistical analysis with two-sided unpaired t-test (B, C), one-way ANOVA analysis (A, D-F), two-way ANOVA analysis (G) and Log-rank test for (H). *P < 0.05; **P < 0.01; ***P < 0.001; ****P < 0.0001; ns, no statistical significance.

Since arbutin does not enhance host immunity against Tg infection, its anti-Tg mechanism likely operates through non-immunological pathways. Considering the critical role of iron in Tg survival [11,12], we investigated arbutin’s regulatory effects on intracellular iron homeostasis using both immune (BMDMs) and non-immune (HFF) cell models. QIP analysis revealed that arbutin treatment significantly increased intracellular QIP levels in both cell types regardless of infection status (Fig 3A), measured at 24 hpi for BMDMs and 72 hpi for HFF cells. QIP elevation signifies a reduction in the labile iron pool (LIP) [13]. This indicates that arbutin mediates depletion of LIP, which serves as a metabolic nexus for both host cells and intracellular parasites.

The cellular iron homeostasis is regulated in part by a balance between transferrin receptor (TFR)-dependent iron uptake and ferroportin (FPN)-dependent iron export [14]. To investigate whether the levels of LIP influence Tg infection outcomes, THP-1 or HFF cells were pre-treated with either a vehicle control or the FPN inhibitor VIT-2763 for 2 hours in the presence of arbutin, followed by infection with Tg RH tachyzoites. The levels of LIP were found to increase after VIT-2763 treatment (Fig 3B), and the relative intracellular parasite numbers increased correspondingly (Fig 3C), suggesting that iron restriction is a crucial defense mechanism against Tg RH infection in both immune and non-immune cells.

Arbutin treatment led to a reduction in TFR1 expression in both Tg RH-infected THP-1 and HFF cells, while its effect on FPN expression varied depending on the cell type (i.e., no change in THP-1 cells; increased in HFFs) (Fig 3D and 3E). Furthermore, the addition of extra iron in vitro reversed the inhibitory effect of arbutin on Tg RH infection (Fig 3F and 3G). Collectively, our data suggest that arbutin inhibits Tg RH infection, at least in part, by limiting intracellular free iron availability.

To confirm the relationship between arbutin and iron in vivo, C57BL/6 WT mice were fed either a high-iron diet (1000 ppm) or a normal-iron (40 ppm) control diet starting on day -7. On day -3, the mice were given either vehicle or arbutin-supplemented drinking water. On day 0, the mice were intraperitoneally infected with Tg RH tachyzoites and continued to receive the same type of diet and water thereafter. We found that the high-iron diet negated the protective effect of arbutin (Fig 3H). Collectively, our data indicate that the anti-*T. gondii* effect of arbutin is associated with its capacity to restrict LIP.

### Arbutin induces heme degrading enzyme HMOX1 expression in macrophages

**Fig 4:**
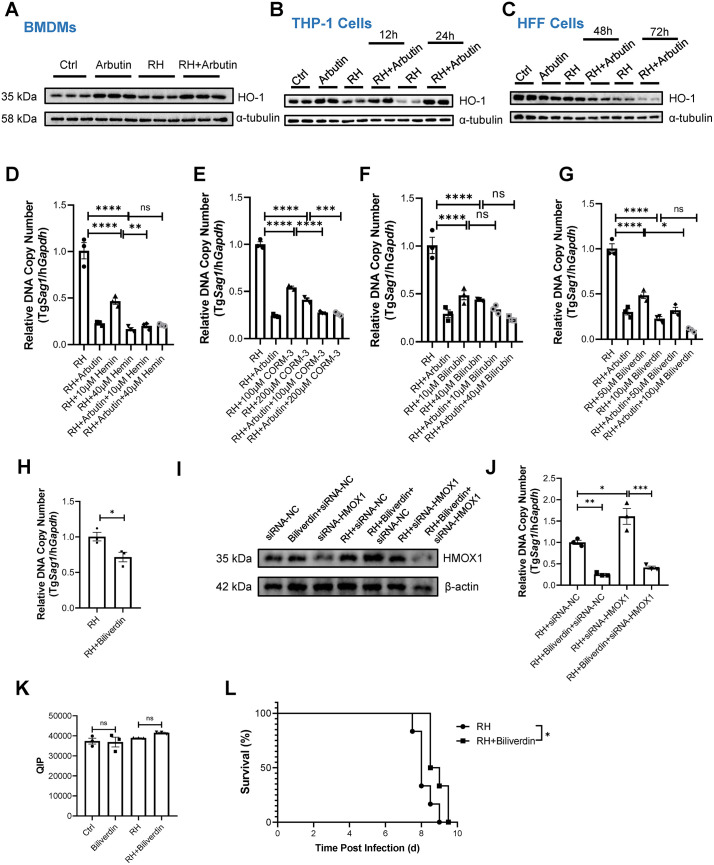
Biliverdin functions as an effector molecule against *T. gondii.* **(A–C)** The expression levels of HMOX1 before or after infection in the presence or absence of arbutin were monitored by immunoblot at 24 hpi for BMDMs, at 12 and 24 hpi for THP-1, and at 48 and 72 hpi for HFFs. n = 3 samples/group. **(D)** The relative intracellular parasite numbers in the presence of hemin with or without arbutin treatment were quantified by qPCR at 24 h post-infection in THP-1 cells. n = 3 samples/group. **(E-G)** The relative intracellular parasite numbers in the presence of CORM-3 (E), bilirubin (F), or biliverdin (G) with or without arbutin treatment were quantified by qPCR at 24 h post-infection in THP-1 cells. n = 3 samples/group. **(H)** The relative intracellular parasite numbers in the presence of vehicle or biliverdin treatment were quantified by qPCR at 72 h post-infection in HFF cells. n = 3 samples/group. **(I and J)** *HMOX1* gene expression was knockdown by siRNA in THP1 cells with or without Tg RH infection and in the presence or absence of biliverdin. (I) The effect of *HMOX1* gene silencing on HMOX1 protein levels was evaluated using immunoblot. (J) The relative parasite loads were determined 24 hpi using qPCR. n = 3 samples/group. siRNA-NC: negative control (NC) scramble siRNA; si-HOMX1: HOMX1-specific siRNA. **(K)** THP-1 cells were incubated with vehicle or biliverdin (50 μM) 2 h before infection with Tg RH. The levels of LIP at 24 hpi were determined using QIP analysis. **(L)** C57BL/6 WT mice were fed a normal control diet (“RH” group) or biliverdin-supplemented diet (20 mg/kg) (“RH + biliverdin” group) starting on day -7. On day 0, the mice were intraperitoneally infected with Tg RH tachyzoites. The survival rate post infection was monitored daily. n = 6 mice/group. The experiments in (A-K) were repeated at least 2 times. Data were shown as the mean ± SEM. Statistical analysis with two-sided unpaired t-test (H), one-way ANOVA analysis (D-G, J, K), and Log-rank test for (L). *P < 0.05; **P < 0.01; ***P < 0.001; ****P < 0.0001; ns, no statistical significance.

Our transcriptomic analysis revealed that arbutin can upregulate the transcriptional level of the heme oxygenase-1 (*Hmox1*) gene in BMDMs (Fig 2B). Additionally, arbutin treatment also increased the transcription levels of several other genes involved in heme biosynthesis and degradation pathways (S1 Fig). Furthermore, we observed that arbutin specifically enhances HMOX1 protein expression levels in macrophage lineages, such as mouse BMDMs and human THP-1 cells (Fig 4A and 4B). In contrast, arbutin suppresses HMOX1 protein expression in fibroblast HFF cells (Fig 4C), indicating a cell-type-specific effect of arbutin on influencing heme metabolic process.

Since a previous study suggests that HMOX1 activity is involved in the control of Tg infection in vivo, but the exact mechanisms remain unknown [15], we explored the possibility of a role of HMOX1 pathway in arbutin-mediated anti-Tg activity in macrophages. THP-1 cells were pre-treated with either vehicle, arbutin, the HMOX1 inducer and activator hemin [16], or a combination of arbutin and hemin, followed by infection with Tg RH. Hemin treatment reduced the parasite load, supporting the role of HMOX1 activity in controlling Tg infection (Fig 4D). However, there was no notable synergistic activity when combined with arbutin (Fig 4D), suggesting that in macrophages arbutin’s anti-Tg RH effect works majorly through HMOX1 pathway.

### The heme degrading intermediate biliverdin functions as an effector molecule against *T. gondii*

HMOX1 catalyzes the oxidative cleavage of the protoporphyrin IX ring in heme, producing biliverdin, carbon monoxide (CO), and labile iron [17]. Biliverdin can subsequently be converted to bilirubin by biliverdin reductase [18]. To investigate the role of heme catabolic intermediates in Tg infection control, we incubated THP-1 cells with either CORM-3 (a water-soluble CO-releasing molecule), bilirubin, or biliverdin, under both arbutin-supplemented and arbutin-free conditions, and then assessed their effects on Tg infection. All tested heme catabolic intermediates independently inhibited Tg growth in THP-1 cells, yet failed to demonstrate notable synergistic activity when combined with arbutin (Fig 4E–4G). Intriguingly, in HFF cells, the anti-parasitic potency of CORM-3 and bilirubin were diminished (S2 Fig), whereas biliverdin maintained inhibitory capacity against Tg infection (Fig 4H). Furthermore, biliverdin maintained its inhibitory effect on Tg growth in THP-1 cells even when HMOX1 expression was attenuated via siRNA knockdown (Fig 4I and 4J), suggesting that biliverdin per se, rather than HMOX1-mediated heme metabolic process, is critical for Tg control. Unlike arbutin, however, biliverdin did not affect the LIP (Fig 4K), suggesting a distinct mechanism of action for biliverdin in combating Tg.

To further validate that biliverdin acts as an effector molecule against Tg, C57BL/6 WT mice were fed either a normal control diet (designated as the “RH” group) or a biliverdin-supplemented diet (20 mg/kg, designated as the “RH + biliverdin” group) starting 7 days prior to infection. On day 0, the mice were intraperitoneally infected with Tg RH tachyzoites. The results showed that the biliverdin-supplemented diet slightly enhanced survival rates post-infection (Fig 4L), thereby providing additional support for biliverdin’s role as an anti-*T. gondii* agent. Collectively, our data indicate that arbutin induces heme degrading enzyme HMOX1 expression in macrophages and the heme degrading intermediate biliverdin functions as an effector molecule against *T. gondii*.

### Arbutin intervention reduced infection-related mortality in immunocompromised mice

**Fig 5:**
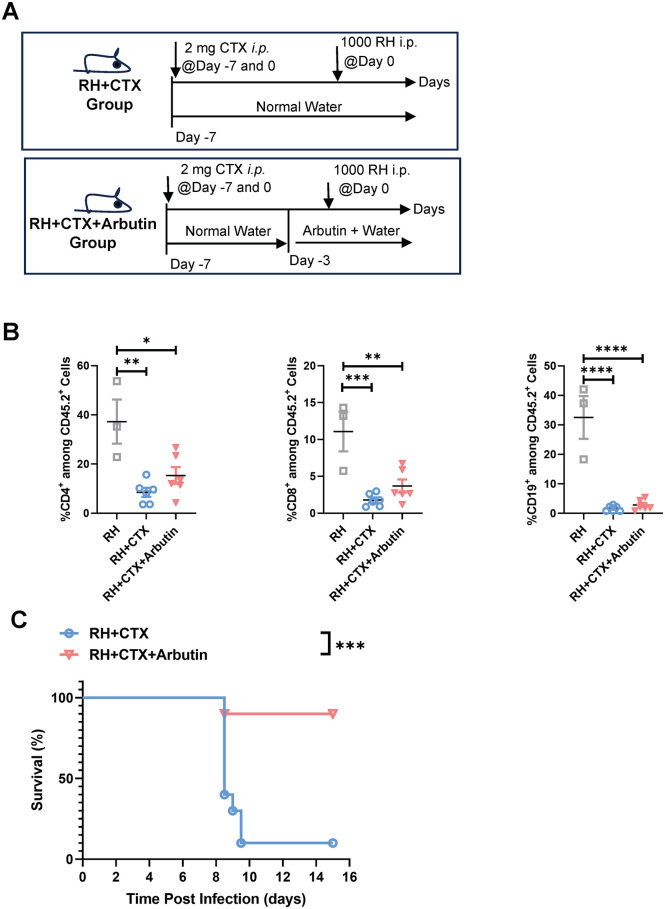
Arbutin intervention reduced infection-related mortality in immunocompromised mice. **(A)** Schematic diagram of the experimental timeline, including cyclophosphamide (CTX) treatment, arbutin supplementation, and primary infection. **(B)** Flow cytometry analysis of the percentage of CD4, CD8, CD19 positive cells among CD45 positive cells in the blood in the indicated groups 7 days post infection. n = 3 (RH group), and 6 (‘RH + CTX’ and ‘RH + CTX + arbutin’ group, respectively). **(C)** Survival rates were monitored daily for 15 days post infection. n = 10 mice/group. Data were shown as the mean ± SEM. Statistical analysis with one-way ANOVA analysis (B), and Log-rank test for (C). ***P < 0.001; ****P < 0.0001.

Based on our evidence that arbutin potentiates cell-autonomous defense while suppressing inflammatory response, we investigated its protective efficacy against Tg infection in immunocompromised hosts. To this end, Kunming WT mice received intraperitoneal weekly injection of cyclophosphamide (CTX) for immunosuppression, starting on day -7 days prior to infection. On day -3, the mice were given either vehicle or arbutin-supplemented drinking water. On day 0, the mice were intraperitoneally infected with Tg RH tachyzoites and continued to receive the same type of water thereafter. Flow cytometry confirmed CTX-mediated depletion of CD4, CD8, CD19-positive hematopoietic cells in blood. Notably, arbutin supplementation significantly improved the survival of the mice post-infection (Fig 5). These findings demonstrate arbutin’s capacity to mitigate Tg-associated mortality even in profoundly immunosuppressed hosts.

### The therapeutic potential of arbutin against chronic infection

Beyond an acute phase which is generally self-limited in immunocompetent hosts, the ability of *T. gondii* to persist as a latent form through bradyzoite-containing cysts is an important aspect of toxoplasmosis. To evaluate the therapeutic potential of arbutin against chronic infection, Kunming WT mice were provided with either vehicle or arbutin-supplemented drinking water for three days prior to intragastric inoculation with 50 cysts of the Wh6 strain, with maintained supplementation throughout the study period. Notably, arbutin administration resulted in significantly reduction of cerebral cyst burden at 29 days post-infection (S3A Fig). Furthermore, in immunocompromised nude mice, arbutin demonstrated a marginal trend toward improved survival outcomes (*P* = 0.0524) (S3B Fig). These findings substantiate arbutin’s therapeutic efficacy against chronic toxoplasmosis.

## Discussion

### Arbutin exerts anti-*Toxoplasma* effects through multiple mechanisms

Our study revealed that the phytochemical arbutin exerts anti-*Toxoplasma* effects through multiple mechanisms: (1) arbutin modulates iron homeostasis to restrict pathogen growth in both immune and non-immune cells, (2) arbutin enhances the biosynthesis of biliverdin, a metabolite with intrinsic anti-*Toxoplasma* activity, in macrophages. However, the exact molecular mechanism by which arbutin modulates intracellular iron homeostasis and biliverdin suppresses *T. gondii* growth requires further investigation. Our preliminary data indicated that arbutin is not able to directly chelate iron in vitro (S4 Fig). Arbutin treatment led to a reduction in TFR1 expression in both Tg RH-infected THP-1 and HFF cells, suggesting that the reduction in the intracellular LIP induced by arbutin is not due to direct iron chelation by the compound itself, but is instead mediated through the remodeling of host cell iron metabolic pathways. Biliverdin not only has a direct antioxidant effect by scavenging free radicals, but also targets many signal transduction pathways, such as soluble guanylyl cyclase and the aryl hydrocarbon receptor [19], all of which may directly or indirectly contributes to its anti-*Toxoplasma* function.

### Inflammation and anti-microbial defense are two discrete functional modules in host defense response

Notably, although inflammation is indispensable for host defense against infection, arbutin paradoxically attenuates *T. gondii* infection-triggered inflammatory responses while maintaining uncompromised parasitical control and host viability. Our data indicate that inflammation and antimicrobial defense represent two discrete functional modules whose activation dynamics need not exhibit positive correlation. Emerging clinical evidence even reveals contexts where chronic inflammation subverts host defense, particularly in obesity-related meta-inflammation states characterized by heightened susceptibility to SARS-CoV-2 infection [20]. These findings collectively underscore the importance of cell autonomous mechanisms in host defense against infection.

### Arbutin doesn’t provide long-term immunity

While arbutin demonstrates efficacy in preventing acute mortality during primary *T. gondii* infection, its therapeutic withdrawal compromises defense against secondary challenge. This vulnerability likely stems from arbutin’s inability to promote inflammation, an essential process required for establishing immunological memory. Our data suggest that inflammation plays a non-redundant role in inducing adaptive immune cell memory, a function irreplaceable by cell-autonomous defense mechanisms. Furthermore, our data highlight a potential limitation for arbutin’s therapeutic use in fighting against Toxoplasmosis (due to lacking of long-term immunity) but also its value in acute hyperinflammatory scenarios.

### The role of HMOX1 in anti-*Toxoplasma* defense in macrophages

Our data demonstrate that arbutin upregulates the expression of HMOX1 in macrophages. HMOX1 facilitates the degradation of free heme, which serves as a vital metabolite for both host and parasite. However, *T. gondii* possesses autonomous heme synthesis capacity to support its intracellular growth and acute virulence [21]. Therefore, host cellular heme deprivation is unlikely to directly impede the growth of *T. gondii*. We did not find any evidence of arbutin in inhibiting the parasite’s heme levels in vitro. Intriguingly, our findings reveal that host heme catabolic intermediates exhibit intrinsic anti-*Toxoplasma* activity. In addition, HMOX1 is able to induce indoleamine 2,3-dioxygenase (IDO), an enzyme involved in tryptophan degradation, in a cell-type-dependent manner [22]. Notably, tryptophan deprivation has been shown to suppress *T. gondii* replication in vitro [23]. The potential requirement of IDO involvement in HMOX-1-mediated anti-*T. gondii* effects warrants future investigation.

### The pharmacologic properties of arbutin may influence its potency in treating Toxoplasmosis

When exposed to high temperature, ultraviolet light, or dilution in an acidic buffer, arbutin in vitro may undergo spontaneous partial hydrolysis to hydroquinone, which can be further oxidized to benzoquinone [24]. This may influence the biological effects of arbutin. Indeed, we had noticed that different batches of arbutin had shown different potency in inhibiting *T. gondii* in vitro (S5 Fig), although these variations do not affect the conclusions of this study. In vivo, arbutin can also be converted to hydroquinone by glycosidases presented in host gut microbiota [25]. Hydroquinone has astringent, disinfectant, and antioxidant properties [26], which may possess anti-parasite activity. However, our preliminary data indicated that hydroquinone is not as effective as arbutin in inhibiting *T. gondii* intracellular growth in vitro (S6 Fig), suggesting that the anti-*Toxoplasma* effect of arbutin does not dependent on its conversion to hydroquinone. While hydroquinone generation in vivo remains a factor for future toxicological studies, the potent anti-parasitic activity we report is likely a direct property of the arbutin molecule, which operates by remodeling host cell metabolism rather than through rapid conversion to its aglycone.

Notably, prolonged exposure to free hydroquinone has genotoxic, carcinogenic, and toxic effects on various organs, including the kidneys and liver, in a species-, strain-, and sex-dependent manner [27]. Hydroquinone has reproducibly produced renal adenomas in male F344 rats [28]. However, humans are routinely exposed to hydroquinone through dietary sources (e.g., cranberries, blueberries, rice, onions, wheat, pears, coffee, tea, and red wine) and the general environment, and studies in which volunteers ingested 300–500 mg of hydroquinone for 3–5 months had no reports of renal or blood abnormalities [29]. Hydroquinone can also be degraded or biotransformed by fungi and bacteria under aerobic or anaerobic conditions [26]. Therefore, host gut microbiota configuration might influence the metabolic flux of hydroquinone and its toxicity to the host. Furthermore, preclinical toxicological evaluation indicates that arbutin may exert a low level of toxicity at high oral doses in mice (LD_50_ = 9804 mg/kg) and rats (LD_50_ = 8715 mg/kg) [30]. In our in vivo studies, the highest concentration of arbutin administered to mice was 50 mg/mL in the drinking water. Based on the average daily water consumption of a mouse (approximately 3–5 mL for a 25 g mouse) and its body weight, we estimated that the daily dosage received by the mouse in the high-dose group to be roughly 6,000–10,000 mg/kg/day.

A single oral dose of 50 mg/ml arbutin administered to a 25-g mouse consuming 0.5 ml arbutin-supplemented water would equate to approximately 1/10 of its LD₅₀ value. Since the LD₅₀ is determined by a single, oral gavage, which results in an immediate high systemic exposure. In contrast, our administration via drinking water allows for voluntary, sporadic intake throughout the day and night. This results in a much slower and sustained absorption, preventing the sharp peak plasma concentrations that cause acute toxicity. Throughout our study, we closely monitored the mice and observed no signs of acute toxicity (e.g., no lethargy, piloerection, or mortality) in any of the arbutin-treated groups prior to infection. This demonstrates that the regimen we used was well-tolerated. In addition, the in vitro dose that we used also induced minimal cell death compared with *T. gondii* infection-induced cell death (S7 Fig). While the therapeutic potential and pharmacologic properties of arbutin remains to be further explored, it at least establishes a precedent for such a dual-action drug to be developed in the future, especially for managing acute infection with hyperinflammation.

## Conclusion

In summary, our work establishes a novel paradigm for combating intracellular parasites by pharmacologically enhancing cell-autonomous defense mechanisms. Arbutin achieves a critical balance in host-pathogen interactions by simultaneously disabling the parasite through nutrient restriction (iron) and effector molecule production (biliverdin), while protecting the host from collateral damage by dampening excessive inflammation. This strategy is effective even in the absence of a functional adaptive immune system, highlighting its potential for treating infections in immunocompromised individuals. Our study provides a potential direction for further development of effective drugs to prevent and treat toxoplasmosis. Future research should employ chemical proteomics or genetic screens to identify the direct protein target of arbutin. Comprehensive long-term in vivo toxicity studies are needed to evaluate the safety of arbutin at the therapeutic doses required for anti-parasitic effects.

## Supporting information

S1 Fig Arbutin treatment increases the expression of key genes involved in heme biosynthesis and degradation pathways in BMDMs. WT BMDMs were pretreated with vehicle or 20 mM arbutin for 12 hours. Cells were then exposed to Tg RH strain tachyzoites (MOI = 1) or left uninfected. At 4 hours post-infection, cells were harvested for RNA sequencing to analyze transcriptomic profiles across experimental groups [“Ctrl”: vehicle, no infection; “Arbutin”: arbutin, no infection; “RH”: vehicle + infection; “Ar_RH”: arbutin + infection]. (A) Schematic of heme biosynthesis and degradation pathways. (B) The heatmap shows the upregulated heme pathway genes by arbutin treatment in the presence or absence of Tg infection.
(TIF)

S2 Fig In HFF cells, the anti-parasitic potency of CORM-3 and bilirubin were diminished. HFF cells were incubated with vehicle, CORM-3 (100 μM or 200 μM), or bilirubin (10 μM or 40 μM) 2 h before infection with Tg RH (MOI = 0.2). The relative intracellular parasite numbers were quantified by qPCR at 72 h post-infection. n = 3. Data were shown as the mean ± SEM. Statistical analysis with one-way ANOVA analysis. ns, no statistical significance.
(TIF)

S3 Fig The therapeutic potential of arbutin against chronic infection. Kunming WT mice or nude mice were provided with either vehicle or arbutin-supplemented (50 mg/ml) drinking water for three days prior to intragastric inoculation with 50 cysts of the Wh6 strain, with maintained supplementation throughout the study period. (A) The number of cysts in the brain of WT mice with or without arbutin treatment at 29 days post-infection. n = 10. (B) The survival curves post infection in the indicated mice. n = 10. Data were shown as the mean ± SEM. Statistical analysis with two-sided Student’s t-test for (A), and Gehan-Breslow-Wilcoxon test for (B). **P < 0.01; ****P < 0.0001; ns, no statistical significance.
(TIF)

S4 Fig Assessment of direct iron-chelating ability of arbutin. (A) A standard curve of iron chelating capacity of EDTA calculated by an ultraviolet-visible spectrophotometric method with a reaction of gallic acid in acetate buffer. (B) The iron chelating capacity of arbutin (5 mM, 10 mM, 20 mM or 50 mM) was determined accordingly. Data were shown as the mean ± SEM. Statistical analysis with one-way ANOVA analysis. ns, no statistical significance.
(TIFF)

S5 Fig Different batches of arbutin had shown different potency in inhibiting *T. gondii* in vitro. WT BMDMs cells were incubated with vehicle or different batch of arbutin (5 mM) 12 h before infection with Tg RH. The relative intracellular parasite numbers were quantified by qPCR at 24 h post-infection. n = 3. Data were shown as the mean ± SEM. Statistical analysis with two-sided Student t-test.
(TIFF)

S6 Fig Hydroquinone is not as effective as arbutin in inhibiting *T. gondii* intracellular growth in vitro. THP-1 cells were incubated with vehicle, arbutin, or hydroquinone (HQ) at the indicated concentration for 12 h before infection with Tg RH. The relative intracellular parasite numbers were quantified by qPCR at 24 h post-infection. n = 3. Data were shown as the mean ± SEM. Statistical analysis with one-way ANOVA analysis. ns, no statistical significance; **P < 0.01; ***P < 0.001.
(TIF)

S7 Fig The cell viability with or without arbutin treatment monitored by CCK8 assay. THP-1 cells were incubated with vehicle or arbutin at indicated concentrations 12 h before infection with Tg RH. The relative cell viability was quantified by CCK8 assay at 24 h post-infection. n = 3. Data were shown as the mean ± SEM.
(TIFF)
