# Supplementary figures and images for: The phytochemical arbutin exerts potent anti-Toxoplasma effects through activation of cell-autonomous defense mechanisms while attenuating inflammation
Source: PLoS Negl Trop Dis. 2025 Dec 11;19(12):e0013815. doi: 10.1371/journal.pntd.0013815 (PMC12711086; doi:10.1371/journal.pntd.0013815)

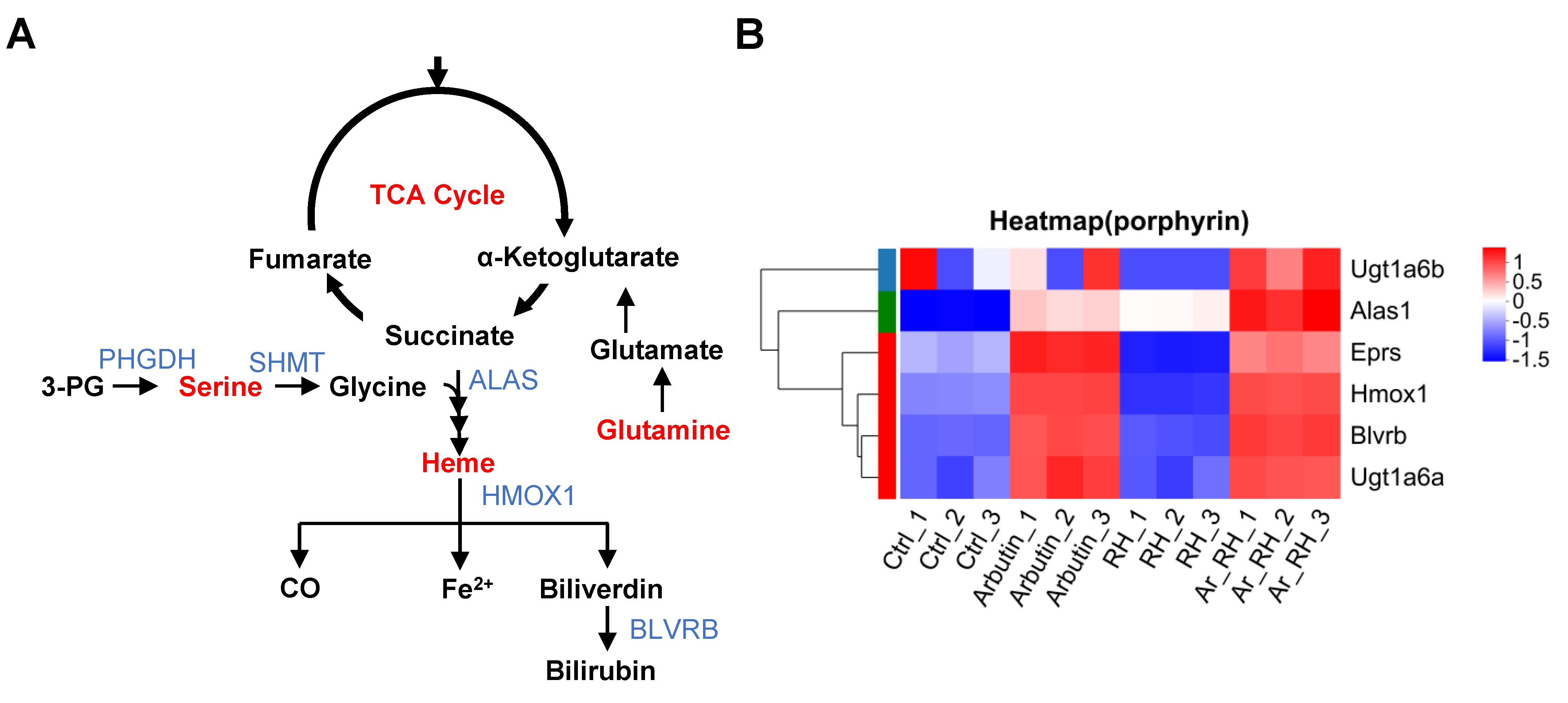

Supplement: S1 Fig — WT BMDMs were pretreated with vehicle or 20 mM arbutin for 12 hours. Cells were then exposed to Tg RH strain tachyzoites (MOI = 1) or left uninfected. At 4 hours post-infection, cells were harvested for RNA sequencing to analyze transcriptomic profiles across experimental groups [“Ctrl”: vehicle, no infection; “Arbutin”: arbutin, no infection; “RH”: vehicle + infection; “Ar_RH”: arbutin + infection]. (A) Schematic of heme biosynthesis and degradation pathways. (B) The heatmap shows the upregulated heme pathway genes by arbutin treatment in the presence or absence of Tg infection. (TIF) [file pntd.0013815.s001.tif]

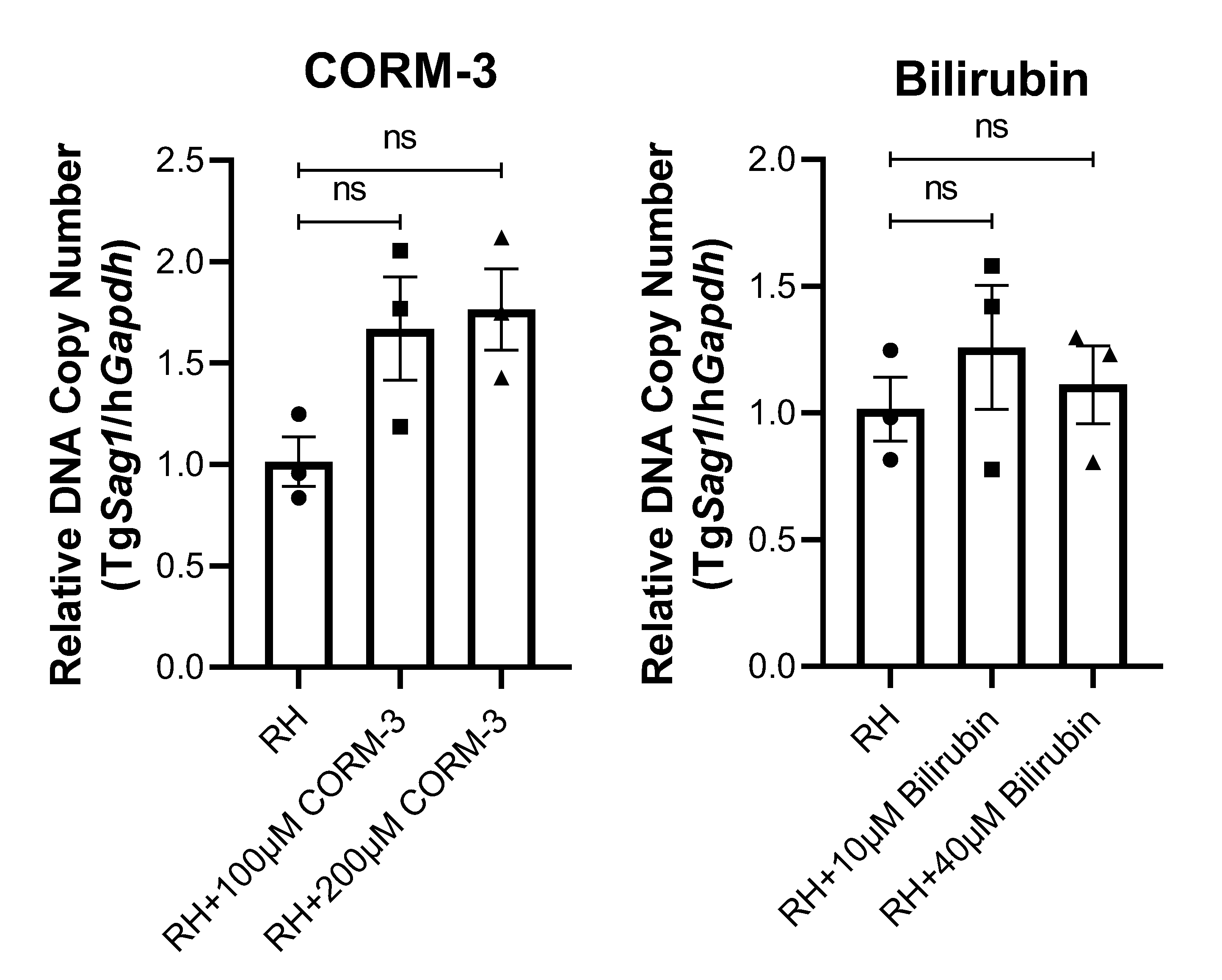

Supplement: S2 Fig — HFF cells were incubated with vehicle, CORM-3 (100 μM or 200 μM), or bilirubin (10 μM or 40 μM) 2 h before infection with Tg RH (MOI = 0.2). The relative intracellular parasite numbers were quantified by qPCR at 72 h post-infection. n = 3. Data were shown as the mean ± SEM. Statistical analysis with one-way ANOVA analysis. ns, no statistical significance. (TIF) [file pntd.0013815.s002.tif]

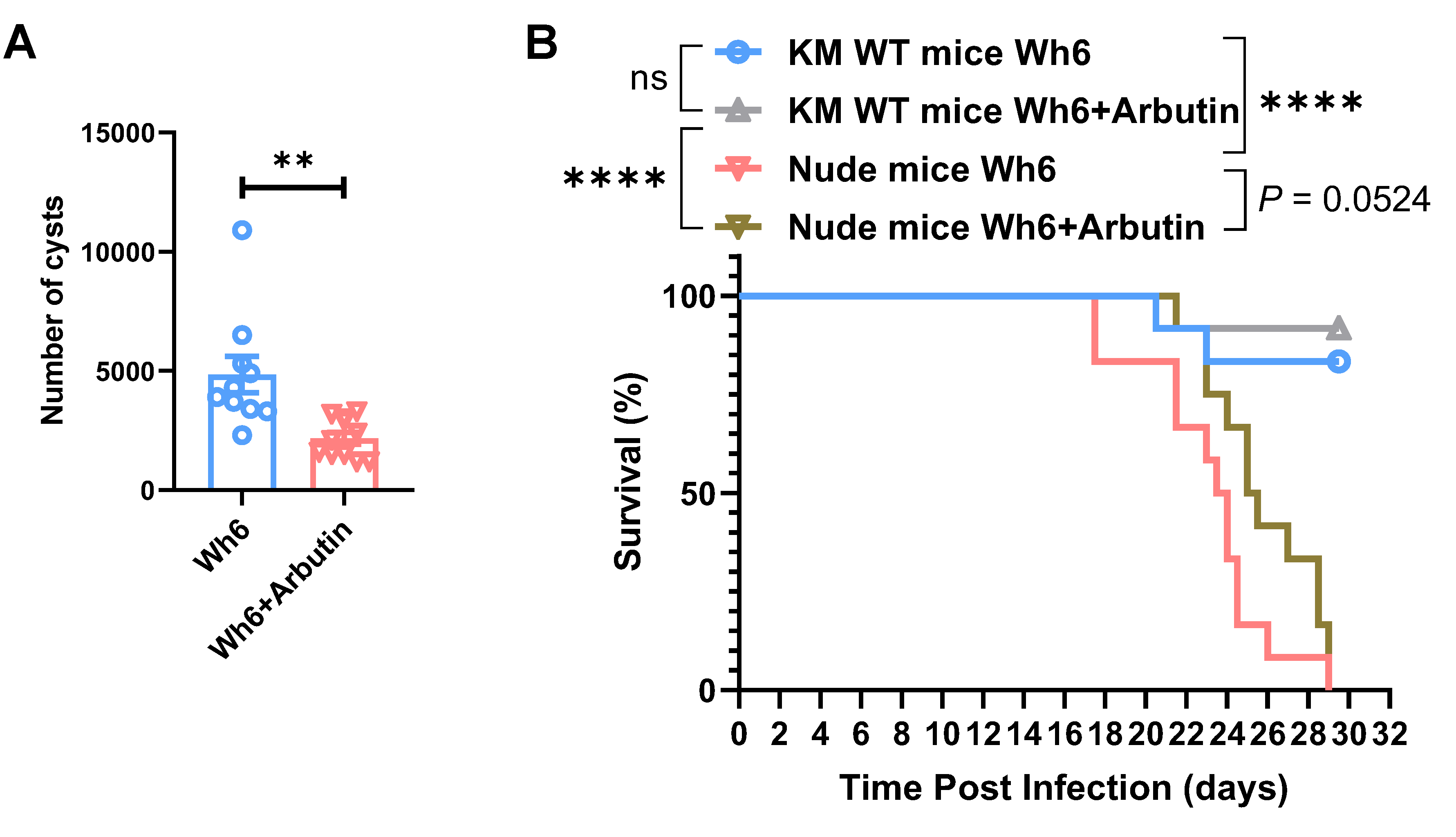

Supplement: S3 Fig — Kunming WT mice or nude mice were provided with either vehicle or arbutin-supplemented (50 mg/ml) drinking water for three days prior to intragastric inoculation with 50 cysts of the Wh6 strain, with maintained supplementation throughout the study period. (A) The number of cysts in the brain of WT mice with or without arbutin treatment at 29 days post-infection. n = 10. (B) The survival curves post infection in the indicated mice. n = 10. Data were shown as the mean ± SEM. Statistical analysis with two-sided Student’s t-test for (A), and Gehan-Breslow-Wilcoxon test for (B). **P < 0.01; ****P < 0.0001; ns, no statistical significance. (TIF) [file pntd.0013815.s003.tif]

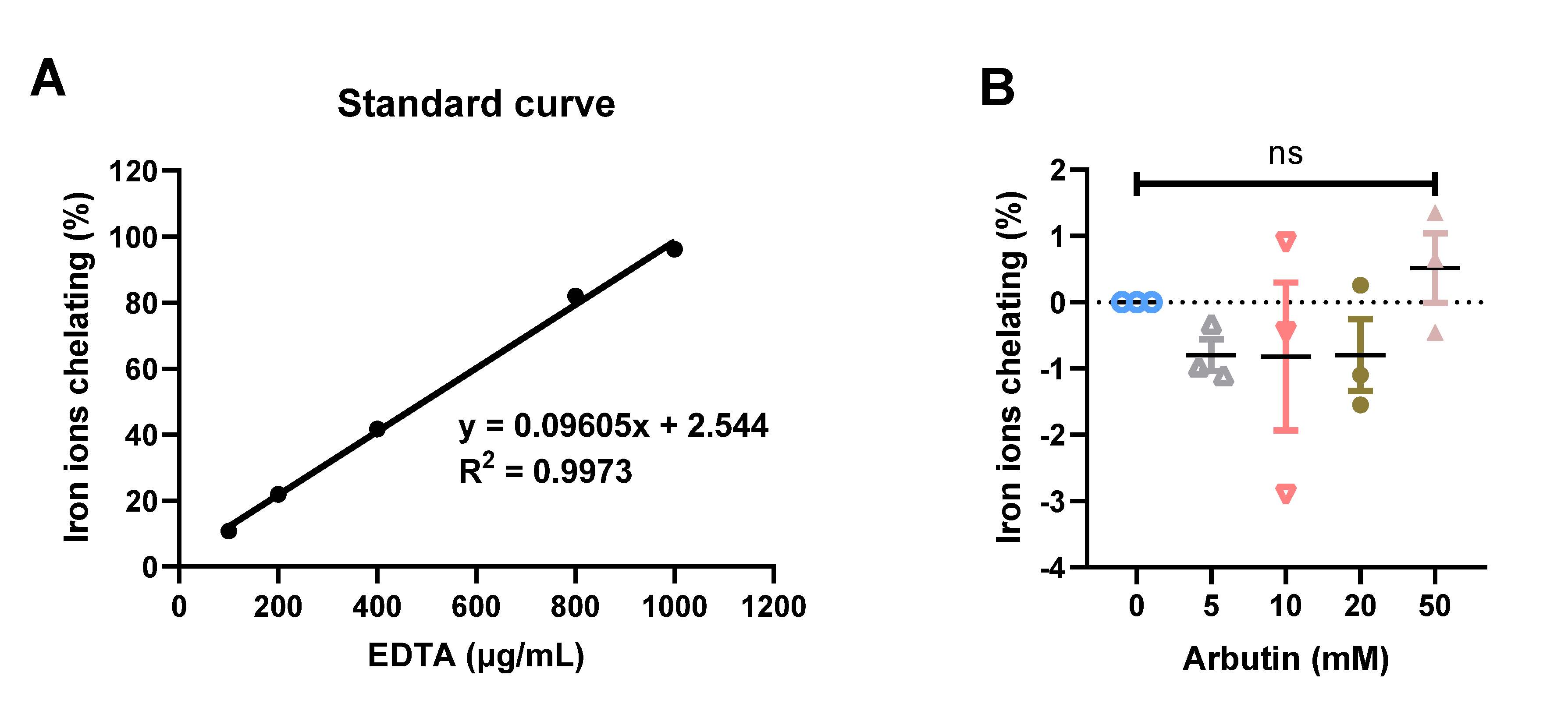

Supplement: S4 Fig — (A) A standard curve of iron chelating capacity of EDTA calculated by an ultraviolet-visible spectrophotometric method with a reaction of gallic acid in acetate buffer. (B) The iron chelating capacity of arbutin (5 mM, 10 mM, 20 mM or 50 mM) was determined accordingly. Data were shown as the mean ± SEM. Statistical analysis with one-way ANOVA analysis. ns, no statistical significance. (TIFF) [file pntd.0013815.s004.tiff]

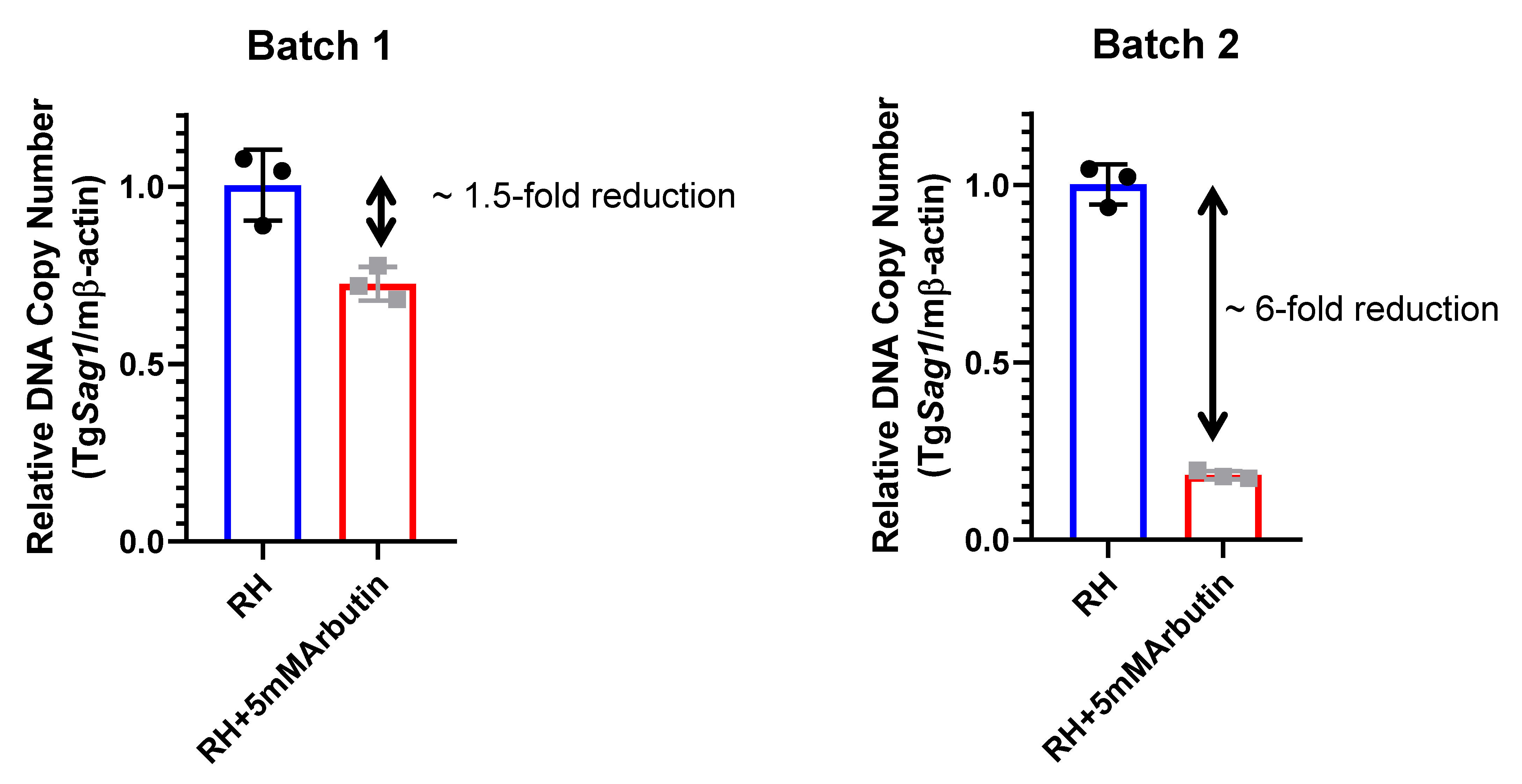

Supplement: S5 Fig — WT BMDMs cells were incubated with vehicle or different batch of arbutin (5 mM) 12 h before infection with Tg RH. The relative intracellular parasite numbers were quantified by qPCR at 24 h post-infection. n = 3. Data were shown as the mean ± SEM. Statistical analysis with two-sided Student t-test. (TIFF) [file pntd.0013815.s005.tiff]

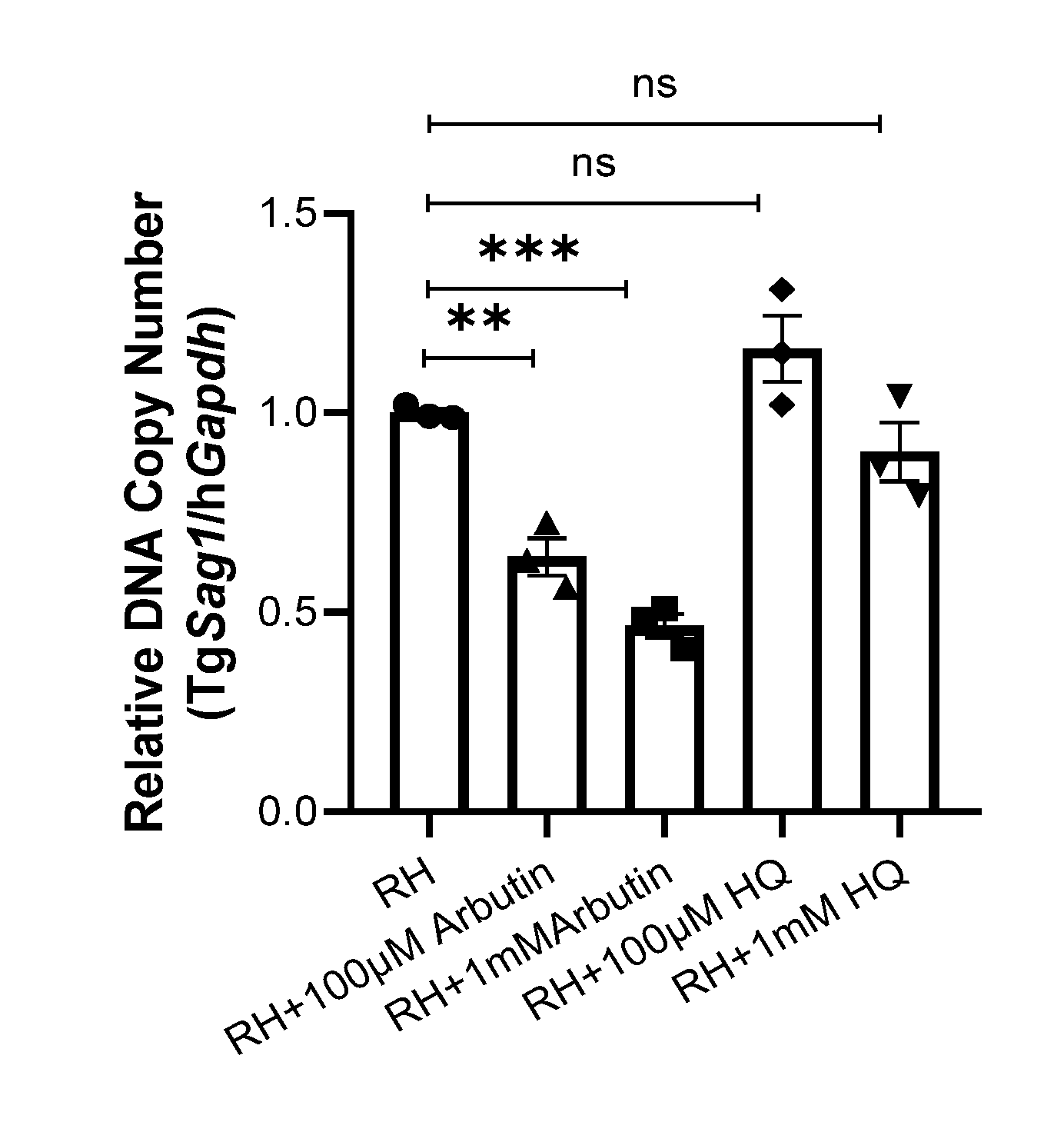

Supplement: S6 Fig — THP-1 cells were incubated with vehicle, arbutin, or hydroquinone (HQ) at the indicated concentration for 12 h before infection with Tg RH. The relative intracellular parasite numbers were quantified by qPCR at 24 h post-infection. n = 3. Data were shown as the mean ± SEM. Statistical analysis with one-way ANOVA analysis. ns, no statistical significance; **P < 0.01; ***P < 0.001. (TIF) [file pntd.0013815.s006.tif]

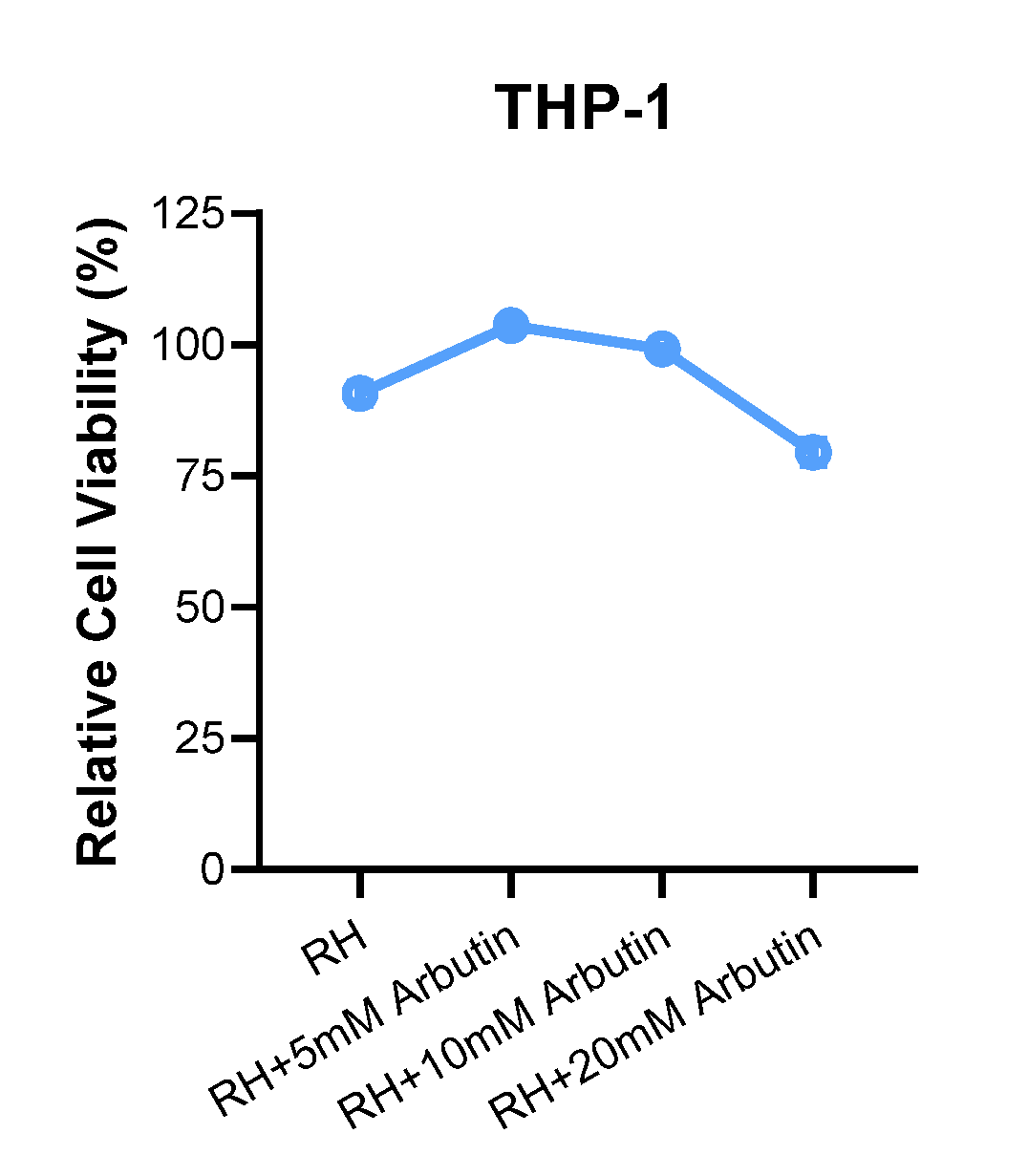

Supplement: S7 Fig — THP-1 cells were incubated with vehicle or arbutin at indicated concentrations 12 h before infection with Tg RH. The relative cell viability was quantified by CCK8 assay at 24 h post-infection. n = 3. Data were shown as the mean ± SEM. (TIFF) [file pntd.0013815.s007.tiff]
